# Beneficial Effects of Sitostanol on the Attenuated Immune Function in Asthma Patients: Results of an In Vitro Approach

**DOI:** 10.1371/journal.pone.0046895

**Published:** 2012-10-16

**Authors:** Florence Brüll, Ronald P. Mensink, Mandy F. Steinbusch, Constanze Husche, Dieter Lütjohann, Geert-Jan Wesseling, Jogchum Plat

**Affiliations:** 1 Department of Human Biology, School for Nutrition, Toxicology and Metabolism, Maastricht University Medical Centre+, Maastricht, The Netherlands; 2 Institute of Clinical Chemistry and Clinical Pharmacology, University of Bonn, Bonn, Germany; 3 Department of Respiratory Medicine, Maastricht University Medical Centre+, Maastricht, The Netherlands; Ludwig-Maximilians-University Munich, Germany

## Abstract

**Background:**

In vitro and animal studies have suggested that plant sterols and stanols increase cytokine production by T-helper-1 cells. This may be beneficial for patient groups characterized by a T-helper-2 dominant immune response, e.g. asthma patients. (1) to evaluate whether sitostanol induces a T-helper-1 shift in peripheral blood mononuclear cells (PBMCs) from asthma patients, and (2) to unravel the role of regulatory T-cells in this respect.

**Methodology/Principal Findings:**

PBMCs from 10 asthma patients and 10 healthy subjects were isolated and incubated with 1.2 µM sitostanol, while stimulated with 5 µg/ml PHA. Similar amounts of cholesterol were used to determine whether effects were specific for plant stanols or for sterols in general. Changes in cytokine production were measured using antibody arrays and ELISAs. Changes in regulatory T-cell population size were measured by flow cytometry, using intracellular Foxp3 staining. Sitostanol increased production of IFNγ by 6.5% and IL-2 by 6.0% compared to cholesterol (p<0.01). No changes in IL-4 and IL-13 were found. Interestingly, this effect was only present in PBMCs from asthma patients. The number of Foxp3+ cells tended to increase and their activity, measured by IL-10 production, increased after sitostanol treatment in PBMCs from asthma patients compared to controls by 32.3% (p = 0.077) and 13.3% (p<0.05), respectively.

**Conclusions/Significance:**

Altogether, the sitostanol-induced Thelper-1 shift in PBMCs from asthma patients and the stimulating effects of sitostanol on Treg cell numbers and activity indicate a possible novel approach for plant stanol ester enriched functional foods in the amelioration of asthmatic symptoms. Functional effects, however, require further evaluation.

## Introduction

Plant sterols are naturally occurring food components with a structural similarity to cholesterol. Plant stanols are the saturated derivates of plant sterols, and both are well known for their serum LDL-cholesterol lowering effects [1], [2]. Moreover, cell culture [3], [4], animal [5], [6], and human intervention studies in different patient groups [7], [8], [9] have suggested that these components have immune modulating properties. In these studies, plant sterols or stanols evoked a T-helper cell (Th)1 response in human or murine T-helper cells, as shown by an increased production of Th1 cytokines IFNγ and IL-2, while the production of specific Th2 cytokines IL-4 and IL-13 remained unchanged. Moreover, we have recently shown that activation of toll-like receptor 2 (TLR2) by plant sterols and stanols is essential in this respect [3].

Asthma is characterized by a disturbed T-helper cell response, i.e. an exaggerated Th2 and an impaired Th1 response, as shown in an *in vitro* model with House Dust Mite (HDM) stimulated peripheral blood mononuclear cells (PBMCs). Stimulated PBMCs from asthma patients clearly showed higher IL-13 and IL-5 and lower IFNγ responses as compared to those of healthy controls [10]. Moreover, it has been demonstrated that this Th2 dominant response is causally related to the incidence of asthmatic symptoms by blocking the IL-13 receptor in CBA/J mice [11]. Therefore it might be beneficial for these patients to shift their skewed Th1/Th2 balance towards a more Th1 dominant response and as such rebalance the disturbed T helper cell system. Indeed, in C3H/HeOuJ mice it has already been shown that stimulating the Th1 response, caused by infection with *Salmonella typhimurium*, reduced symptoms belonging to a Th2 dominant allergic response [12]. Moreover, in line with our observation that plant sterols and stanols act via TLR2, Aumeunier and coworkers have shown in an elegant series of experiments that systemic TLR2 activation suppressed experimental allergic asthma in NOD mice [13]. Also, based on these observations, it was tempting to suggest that the plant stanol induced Th1 shift could also positively influence the function of immune cells, and more particularly the T-helper cells in asthmatic patients.

Besides the above-described skewed Th1/Th2 balance, it has also been suggested that a disturbed balance between Regulatory T-cells (Treg) and Th17 cells may play a role in asthma. Regulatory T-cells are able to suppress the activity of either T-helper cell subtype, i.e Th1, Th2 or Th17, and are therefore important modulators of immune function. Th17 cells are characterized by the production of pro-inflammatory cytokines IL-17a and IFNγ, and play a major role in inflammatory and autoimmune diseases [14], [15], [16]. It has been shown that asthma patients have lower numbers of Treg cells with an impaired activity [17], [18], [19] and the importance of IL-10 stimulation in the amelioration of asthmatic symptoms is already well established [20], [21]. Additionally, IL-17a concentrations in bronchoalveolar lavage (BAL) fluid from asthma patients are elevated [22]. Effects of plant stanols on Treg or Th17 cell types have not yet been reported and need to be evaluated.

Recently, the newly described T-cell subtype Natural Killer T (NKT) cells has gained substantial interest regarding their role in asthma pathology [23], [24], [25], [26], [27] NKT cells are found in increased amounts in BAL fluid from patients with severe and poorly controlled asthma [28] and are thought to produce high amounts of IL-4 and IL13 in response to allergens, and of IL-13 and IL-17a in response to viral infections and ozone, respectively. It is now of interest to evaluate the effects of plant stanols on counts and behaviour of this recently defined hybrid cell population in asthma patients.

Therefore, the aims of the study described here were 1) to characterize the differences in immune response between PBMCs isolated from asthma patients and healthy controls, 2) to evaluate whether plant stanols induce Th1 specific cytokine production in PBMCs isolated from asthma patients, and 3) to explore whether the effects of plant stanols are specific for Th1 and Th2 cells, or that these compounds influence behaviour of other immune cells, i.e. Treg, Th17, NKT and NK-cells as well.

## Methods

### Subjects and design

Ten asthma patients and ten apparently healthy control subjects, matched for sex, age and BMI, were enrolled in the study. They were recruited using flyers in the university and hospital buildings and by advertisements in local newspapers. People with inflammatory diseases such as Inflammatory Bowel Disease, autoimmune diseases, diabetes, other lung-related diseases, or who had been on immunosuppressive drug therapy were not allowed to participate. Smoking was prohibited and also the use of plant sterol or plant stanol enriched food products was not allowed. Asthma patients were defined by the self-reported daily use of prescribed medication specifically aimed at the relief of asthmatic symptoms and all participants were using either salmeterol/fluticasone (seretide), budesonide/formoterol (symbicort), salbutamol (ventolin), or a combination of these. All patients had to be using the same prescribed medication and the same dosage for the last six months to ensure their asthma was in a well-controlled state. Prednisone users were excluded, as this medicine suppresses T-helper cell function, which might potentially bias the study outcomes. All patients reported history of allergic asthmatic reactions and participants with exercise-induced asthma were excluded. Based on self-reporting, the healthy control subjects did not have any allergic or asthmatic symptoms.

Each participant had to visit the university twice for fasting blood collections. A standardized lasagne meal was consumed the evening before the blood collection in order to minimize differences in diet-induced variation in T-helper cell behaviour between the two test days. In addition, subjects were not allowed to eat or drink after 10 p.m. Only water and tea without sugar or milk were allowed on the morning of the blood collection. There was a four-week period in between the two blood collections to prevent variations in outcomes due to hormonal changes in female subjects. During both visits a food frequency questionnaire had to be filled out, to check whether dietary habits were comparable in the 4 weeks preceding the two measurements. The study was approved by the Medical Ethics Committee of the Maastricht University Medical Centre+ and all participants have given written informed consent before the start of the study.

### Blood sampling

Blood was collected after an overnight fasting in 5 mL serum tubes (BD Vacutainer) for the measurement of cholesterol, triglyceride, and plant sterol concentrations; in 4 mL EDTA tubes (BD Vacutainer) for the measurement of circulating cytokine concentrations; and in 8 mL sodium-heparine tubes (BD Vacutainer) for the isolation of PBMCs to be used in cell culture experiments. Serum tubes were kept at room temperature for at least 30 minutes, until the blood was completely coagulated. Tubes were then centrifuged at 1300*g for 15 minutes at room temperature. After centrifugation, serum was directly stored at −80°C until further analysis. EDTA tubes were kept on ice and centrifuged within 30 minutes after sampling at 1300*g for 15 minutes at 4°C. The plasma was immediately snap frozen and stored at −80°C. Sodium-heparine tubes were kept on ice and used for cell culture within 60 minutes.

### Lipid, lipoprotein, lathosterol and plant sterol analyses

Concentrations of total cholesterol (Roche Diagnostics Corporation), HDL cholesterol (precipitation method; Roche Diagnostics Corporation), and triglycerides with correction for free glycerol (GPO Trinder; Sigma-Aldrich) were analyzed enzymatically in all serum samples. Serum LDL cholesterol concentrations were calculated using the Friedewald formula [29]. Serum plant sterol (sitosterol and campesterol) and cholesterol precursor (lathosterol) concentrations were measured using GC-MS as described earlier [30].

### Cell culture

Directly after blood collection, PBMCs from all participants were freshly isolated from cold sodium-heparin tubes using lymphoprep (Nycomed) gradient centrifugation as indicated by the manufacturer. Cells were cultured in RPMI 1640, containing 25 mM HEPES and L-glutamine (Gibco), supplemented with 1% penicillin/streptomycin, 1% sodium pyruvate and 1% human serum pool, heat inactivated for 30 minutes at 56°C. PBMCs were seeded 1*10^6^/mL in 24-wells flat bottom culture plates (Corning) and stimulated with 5 µg/mL PHA (Roche) to induce T-cell proliferation. 1.2 µM sitostanol (Sigma), mimicking the physiological serum concentration after dietary plant stanol ester supplementation, or cholesterol (Sigma) was added to the cells, using 2 mM 2-hydroxypropyl-beta-cyclodextrin (CD) (Sigma) as a carrier. For this, sitostanol and cholesterol stock solutions in ethanol were evaporated under N_2_ and re-dissolved in CD. As carrier control condition, 2 mM CD was used. The same experiments were done with 10 ng/mL HDM stimulation instead of PHA stimulation, in order to evoke an allergic response in T-helper cells. After 52 hours PHA stimulation or 7 days HDM stimulation, culture medium of the stimulated PBMCs was removed and stored at −80°C until further analysis

### Treg population size

In order to evaluate effects of plant stanols on the Treg population size, freshly isolated PBMCs were cultured as mentioned above, stimulated with PHA and harvested after 48 hours. Cells were labelled with FITC-αCD4, PE-αFoxp3 and APC-αCD25 (eBioscience), using the eBioscience Foxp3 staining kit. Foxp3 population was characterized as CD4+/CD25++/Foxp3+ and expressed as percentage of total lymphocytes. Measurements were carried out on a FACS CANTO II (Beckton&Dickinson) and analyzed with free WinMDI 2.9 software.

### Cytokine and IgE measurements

Cytokine concentrations in the cell culture supernatant of the PHA stimulations were analysed using a 4-plex custom cytokine electroluminescence array (MesoScaleDiscovery), containing IL-2, IL-4, IL-10 and IL-13. For the determination of IFNγ and IL-17a in culture medium sandwich ELISAs were used (eBioscience). The supernatant from the HDM stimulation was analysed for the presence of IFNγ and IL-13 using sandwich ELISAs (eBioscience). Finally, cytokine concentrations in fasting plasma were measured using a 7-plex custom cytokine electroluminescence array (MesoScaleDiscovery), which included a cytokine detection panel consisting of IFNγ, IL-2, IL-4, IL-10, IL-12p70, IL-13 and IL-17a. Plasma total IgE concentrations were measured with a sandwich ELISA (eBioscience).

### NK and NKT cell population and activity

Freshly isolated PBMCs were cultured in the presence of cholesterol or sitostanol for 16 hours in the presence of HLA-free K562 target cells. NK and NKT cells that degranulate while lysing these HLA-free K562 target cells express the CD107a surface marker [31], which was measured by flow cytometry. For this we used the antibodies APC-αCD3 (eBioscience) and PE-αCD56 (eBioscience) for the characterization of NK cells (CD3−/CD56+) and NKT cells (CD3+/CD56+) in combination with FITC-αCD107a (Beckton&Dickinson). Results were measured using the FACS CANTO II (Beckton&Dickinson) and analyzed with WinMDI 2.9.

### Statistics

For each culture condition, the effects on cytokine production of cholesterol and sitostanol were expressed as the change relative to those of the carrier control. These changes were not normally distributed as indicated by the Shapiro-Wilk test. Effects of cholesterol, sitostanol, as well as the difference in effects of these two sterols, were therefore tested using the Wilcoxon signed rank test. For cell counts, the same comparisons were made, but now a paired t-test was used since data were normally distributed. Next, differences in changes in cytokine concentrations and cell counts from asthma patients and healthy controls were compared using a Mann-Whitney U test and an independent t-test, respectively. Spearman rank correlation coefficients were calculated to examine relationships between effects. P-values less than 0.05 were considered to be statistically significant and p-values between 0.05 and 0.10 were regarded as a trend. All statistical analyses were performed using SPSS16 software.

## Results

### Subject characterization

Baseline characteristics of the subjects are shown in table 1. No smokers were included in the study and all women were pre-menopausal. At the mornings of the blood collections, subjects used their regular medication, which was identical for both days. The subjects of either group did not change their dietary habits between the two blood collections and no differences in cytokine responses between the two days were found (data not shown).

**Table 1:** Baseline subject characteristics.

	Healthy controls	Asthma patients
*M/F*	3/7	3/7
*Age (years)*	32±10 (range 18–51)	32±10 (range 18–52)
*BMI (kg/m^2^)*	25.4±2.9	25.7±2.9
*Cholesterol (mmol/L)*	5.64±0.99	5.84±0.89
*LDL-C (mmol/L)*	3.43±1.00	3.45±0.67
*HDL-C (mmol/L)*	1.55±0.36	1.77±0.49
*Triglycerides (mmol/L)*	1.47±0.49	1.35±0.60
*Sitosterol (*µ*mol/L)*	5.81±0.16	5.37±0.08
*Campesterol (*µ*mol/L)*	9.65±0.20	9.02±0.12
*Lathosterol (*µ*mol/L)*	8.03±0.18	7.07±0.07
*Seretide/Symbicort/Ventolin*	0/0/0	1/8/5

No significant differences were found in characteristics like Treg and NKT activity or population size between asthma patients and controls. Remarkably, asthma patients had a significantly higher percentage of circulating NK cells: 9.66% compared to 2.17% in the control group (p<0.05), as shown in table 2. CD107a expression on these cells, however, did not differ. Cytokine concentrations in fasting plasma were under the lowest limit of detection of the assay.

**Table 2:** Baseline cell counts and CD107a expression.

	Healthy controls	Asthma patients
*Treg (%)*	7.53±3.74	9.17±4.60
*NK (%)*	2.17 (5.19)	9.66 (14.08)*
*CD107a expression on NK cells (%)*	11.00 (17.76)	8.18 (10.57)
*NKT (%)*	2.27 (3.89)	2.69 (3.70)
*CD107a expression on NKT cells (%)*	24.21 (42.70)	21.93 (32.33)

Healthy controls and asthma patients did not differ at baseline, apart from the percentage of NK cells in the PBMC population where asthma patients had a significantly higher NK cell count (9.66% v 2.17%, p<0.05). Data are presented as mean ± SD or median (range).

* p<0.05.

**Figure 1:**
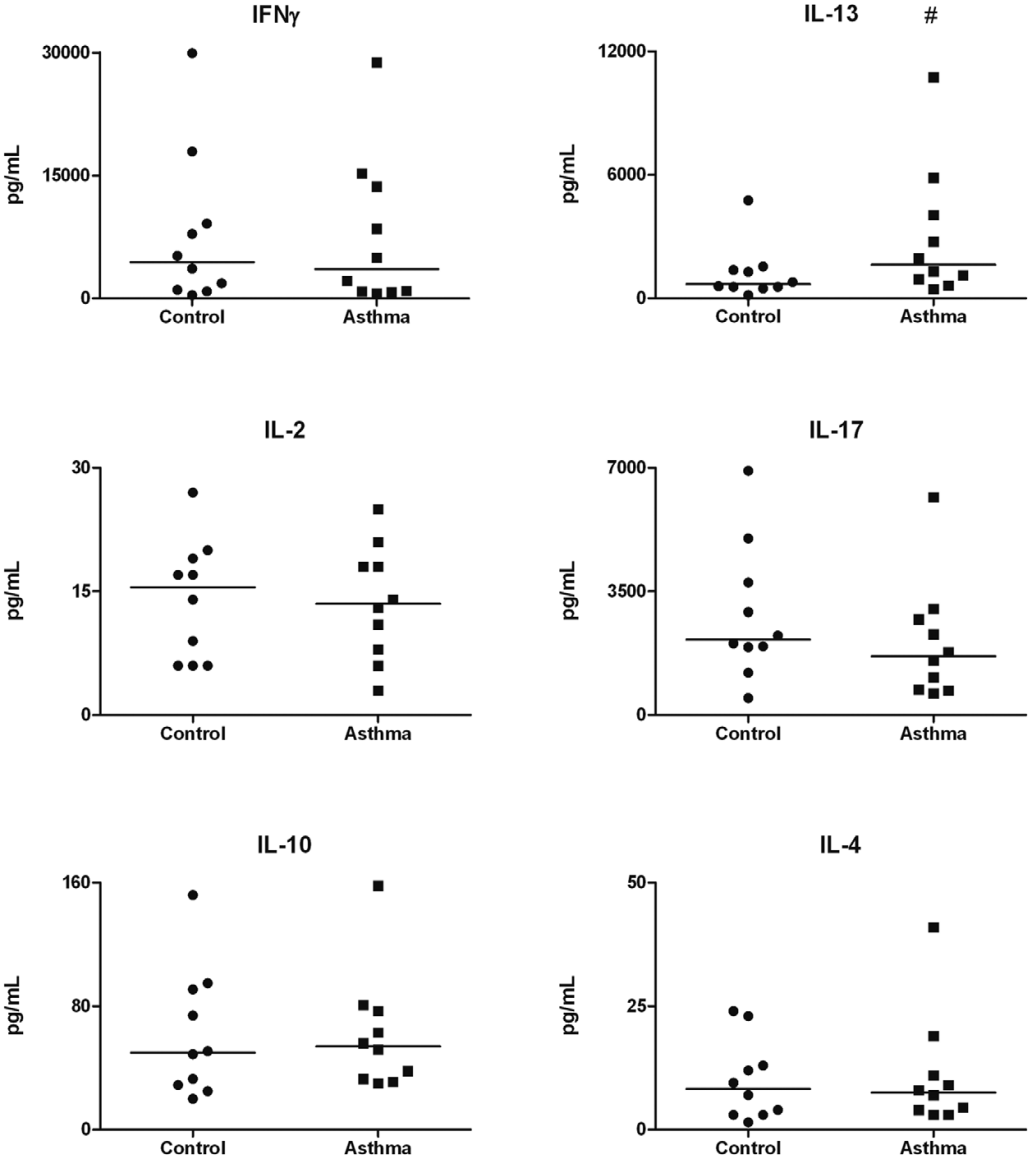
Cytokine responses upon stimulation with a purified House Dust Mite extract. PBMCs from asthma patients tended to produce more IL-13 than cells from healthy controls (1632 pg/mL v 695 pg/mL, p = 0.096). Other cytokines did not differ between asthma patients and healthy controls. Data are presented as single values and medians. # p<0.10.

Upon stimulation with a purified HDM extract, PBMCs from asthma patients tended to have a higher IL-13 response (1632 pg/mL) than healthy controls (695 pg/mL p = 0.096), suggesting a Th2 skewed response in asthma patients. For the production of cytokines IFNγ, IL-2, IL-10, IL-17 and IL-4, no differences were found (figure 1),

### Cytokine responses to sitostanol

**Figure 2:**
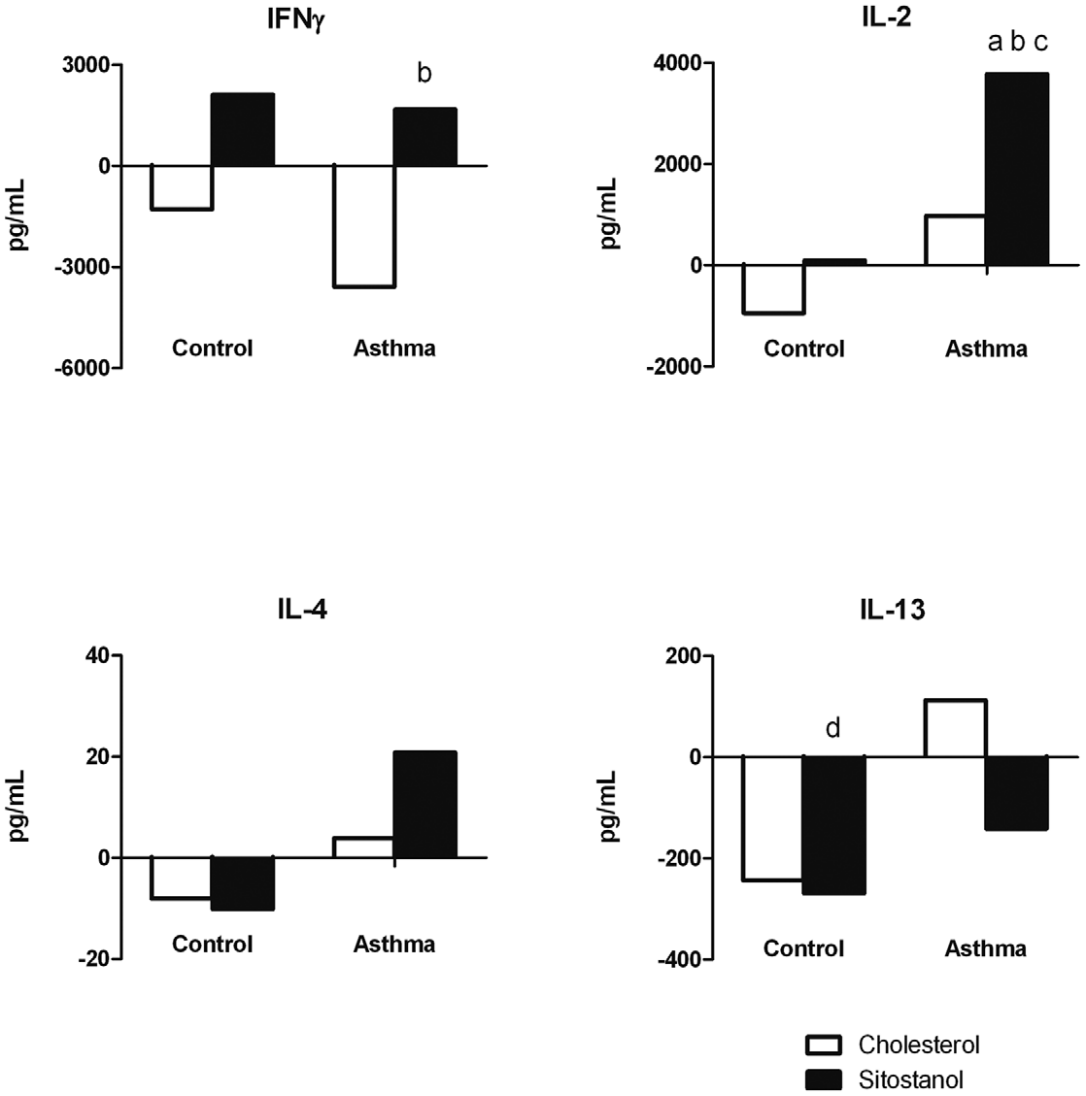
Effects of cholesterol and sitostanol on cytokine production in PBMCs. Sitostanol increased the IL-2 production in asthma patients when compared to cyclodextrin, cholesterol and healthy controls (p<0.05). Data are presented as median changes to the cyclodextrin condition. a = p<0.05 v cyclodextrin; b = p<0.05 v cholesterol; c = p<0.05 v healthy controls; d = p<0.10 v cyclodextrin.

As shown in figure 2, cholesterol did not alter the IFNγ production in PBMCs from asthma patients and healthy controls. Sitostanol significantly increased IFNγ production by 4493 pg/mL (p<0.05) when compared to cholesterol, but only in PBMCs from asthma patients. However, this effect was not seen when the sitostanol condition was compared to cyclodextrin, indicating that the observed effect was merely caused by a decrease in IFNγ production in the cholesterol condition. and there were no significant differences between the effects in asthma patients and healthy controls.

Production of Th1 cytokine IL-2 was also not affected by cholesterol incubation, but sitostanol significantly increased IL-2 production by 3784 pg/mL (p<0.05) when compared to cyclodextrin and by 2101 pg/mL when compared to cholesterol (p<0.05). Again, these effects were only found in cells from asthma patients. The effects of cholesterol and sitostanol differed significantly between the groups (both p<0.05), but the difference between cholesterol and sitostanol did not vary between asthma patients and healthy controls.

No effects of cholesterol or sitostanol were found on the production of Th2 cytokines IL-4 and IL-13 in either asthma patients or controls, with the only exception for sitostanol, which tended to decrease IL-13 production in the control group when compared to the cyclodextrin condition (−268 pg/ml p = 0.093).

This apparent Th1 shift was supported by correlations between cytokine responses. In PBMCs from the control group the production of IFNγ and IL-4 in response to sitostanol correlated significantly (ρ = 0.806 p<0.05), suggesting a balanced response. However, in PBMCs from asthma patients the increase in IFNγ correlated significantly with IL-2 (ρ = 0.648 p<0.05), but not with IL4 or IL-13, showing a Th1 dominant shift in response to sitostanol. In the HDM cultures, no significant cholesterol or sitostanol effects were found (data not shown). Subdividing the asthma group in allergic and non-allergic (i.e. circulating total IgE >100 or <100 U/L) did not affect the results, as the same parameters differed significantly in both subgroups.

### Regulatory T-cell counts and activity, and Th17 activity

After 48 hours of incubation with sitostanol, the PBMC population from asthma patients tended to contain more Treg cells than the cells from healthy controls. The change in Treg cell counts was 10.4% and −14.4% for asthma patients and healthy controls respectively (p = 0.077). However, this effect was only found when sitostanol was compared with cyclodextrin and not with cholesterol. Cholesterol had no effects on Treg cell counts in either group when compared to cyclodextrin.

**Figure 3:**
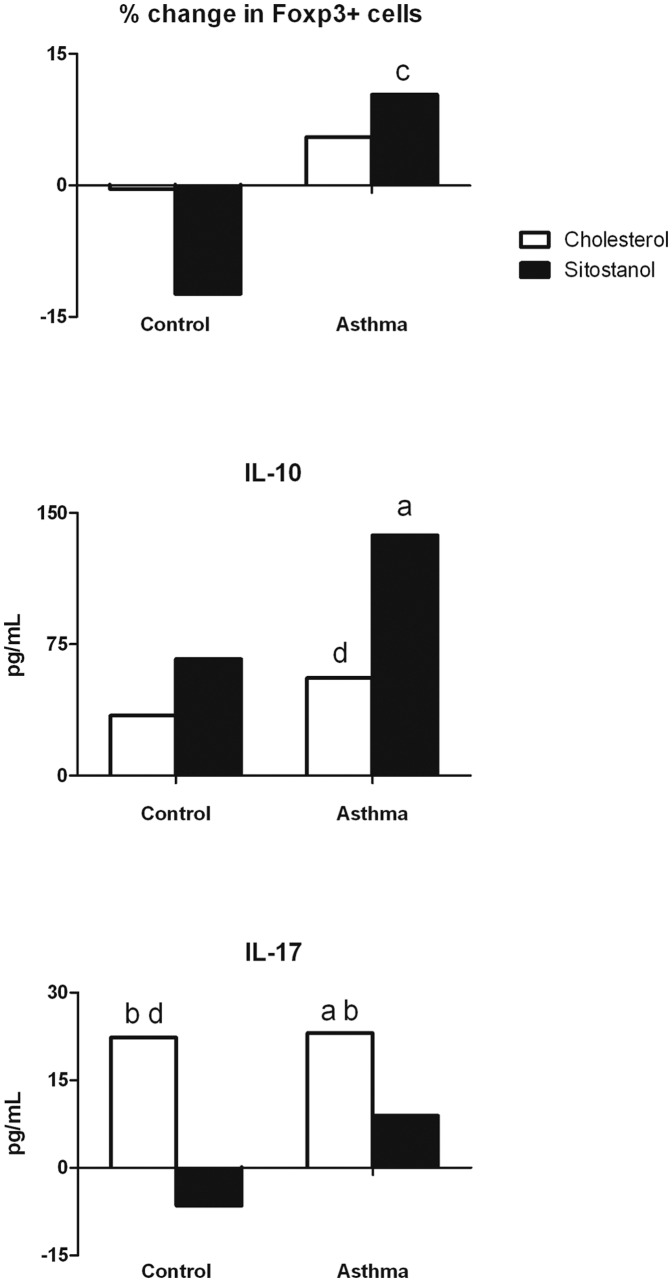
Effects of sitostanol and cholesterol on the percentage of Treg cells and the production of IL-10 and IL-17. Sitostanol tended to induce a higher percentage of Treg cells in the total PBMC population from asthma patients (p = 0.77), and significantly increased IL-10 production (p<0.05). Cholesterol significantly increased IL-17 production when compared to sitostanol (p<0.05). a = p<0.05 v cyclodextrin; b = p<0.05 v sitostanol; c = p<0.10 v healthy controls; d = p<0.10 v cyclodextrin.

After 52 hours of incubation, cholesterol tended to increase the production of Treg cytokine IL-10 (56 pg/mL p = 0.074) and for the sitostanol condition this increase reached statistical significance (137 pg/mL p = 0.047). However, this effect was only seen in cells from asthma patients, and effects of cholesterol and sitostanol did not differ significantly from each other and no significant differences between asthma patients and healthy controls were found (figure 3).

No effects of sitostanol were found on IL-17a production, but a significant increase in IL-17a production was seen after cholesterol was added to the cells from asthma patients, when compared to cyclodextrin and to sitostanol (23 pg/mL p<0.05 and 18 pg/mL p<0.05, respectively). In healthy controls a similar pattern was found when compared to sitostanol (35 pg/mL, p<0.05), but when compared to cyclodextrin it only reached borderline significance (22 pg/mL, p = 0.074). In the control group, the difference between cholesterol en sitostanol tended to be larger than in the asthma patients (p = 0.07) (figure 3).

### NK and NKT cell activity

**Figure 4:**
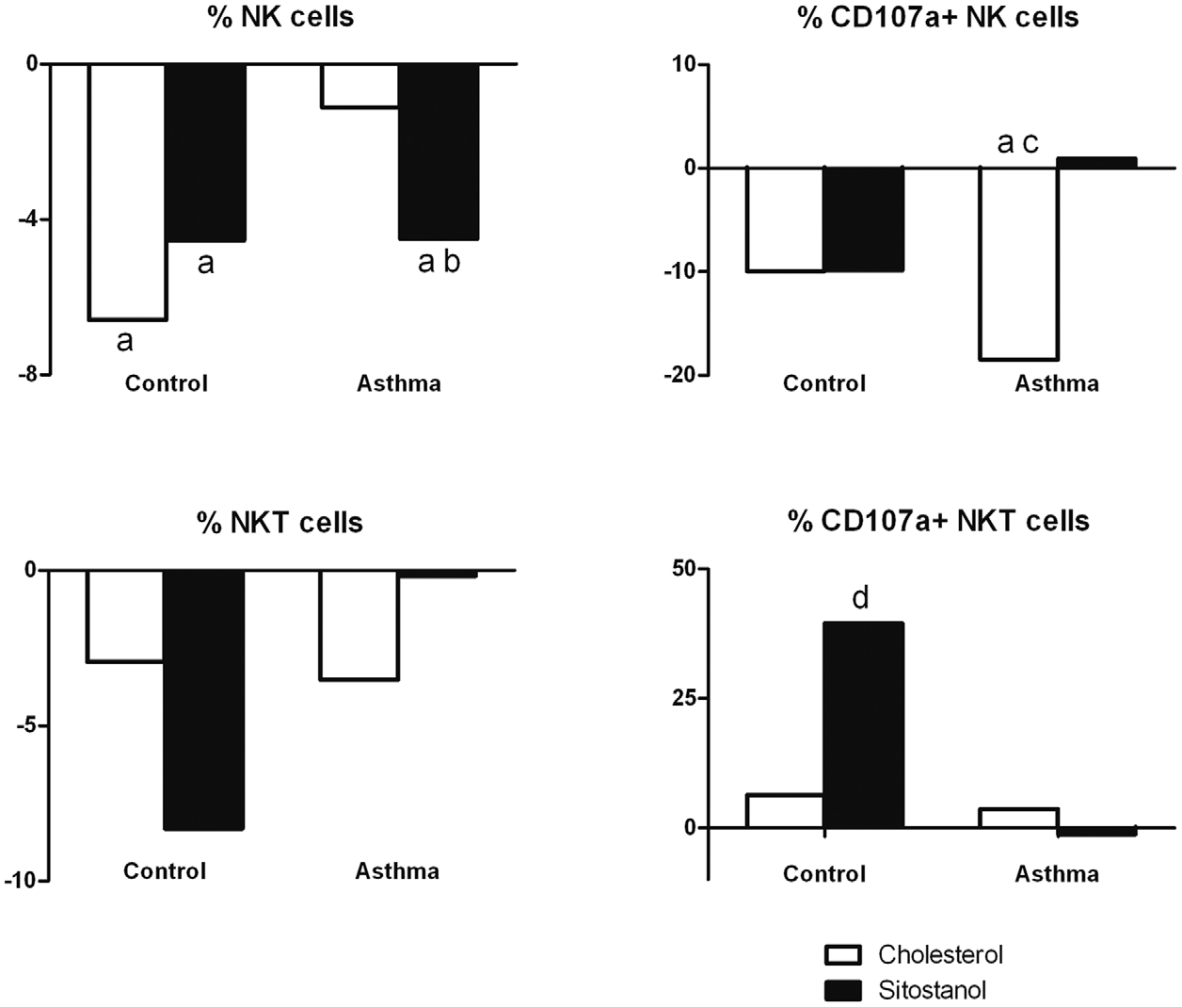
Effects of sitostanol and cholesterol on NK cells, NKT cells and CD107a expression after exposure to K562 cells. Sitostanol significantly reduced the percentage of NK cells in asthma patients as well as in healthy controls (p<0.05). Cholesterol lowered the activity of NK cells in asthma patients (p<0.05) and sitostanol did not increase the activity of NKT cells in asthma patients, but only in healthy controls (p<0.05). a = p<0.05 v cyclodextrin; b = p<0.10 v cholesterol; c = p<0.05 v sitostanol; d = p<0.10 v asthma patients.

Adding sitostanol to the cell culture significantly decreased the population size of NK cells in asthma patients when compared to cyclodextrin. The effect was similar when compared to cholesterol, but only reached borderline significance (p = 0.074). In healthy controls, sitostanol as well as cholesterol significantly decreased the number of NK cells. Notably, sitostanol did not affect NK cell activity in PBMCs from asthma patients. Cholesterol, however, decreased the expression of CD107a significantly in PBMCs from asthma patients (18% and 21% when compared to cyclodextrin and sitostanol respectively, p<0.05), but this was not seen in healthy controls (figure 4).

Neither sitostanol nor cholesterol did alter the number of NKT cells in either PBMCs from asthma patients or from healthy controls. However, the activity of NKT cells tended to be reduced in asthma patients after the addition of sitostanol, when compared to the healthy controls (p = 0.06) (figure 4).

## Discussion

It has been suggested that plant sterols and stanols can specifically increase the activity of Th1 cells [5], [8], [9]. The challenge now is to define conditions in which these effects may be applied functionally. Since asthma patients are characterized by a Th2 dominant immune response, stimulation of the Th1 cells might ultimately be beneficial in counteracting asthmatic symptoms. In this respect, in an allergy model in C3H/HeOuJ mice, it has already been shown by Eigenmann and colleagues [12] that stimulation of Th1 cells, caused by a fungus infection, relieved the mice from their allergic symptoms. Also, blocking the Il13 receptor improved asthma in CAB/J mice [11], indicating the pathologic role of this specific Th2 cytokine [32]. Very recently, it has been shown that beta-sitosterol has the ability to reduce airway inflammation in an asthma model in guinea pigs [33]. Fully in line with this finding, we here show that sitostanol induced a Th1 response in PBMCs from asthma patients, indicated by an increase in the concentration of cytokines IL-2 and IFNγ. This Th1 shift was specific for asthma patients, as no significant effects were found in the healthy controls. In our short-term in vitro experiments, we did not find any effects of sitostanol on the production of Th2 cytokines IL-4 and IL-13. We did not find differences between asthma patients with high and normal total IgE plasma concentrations, possibly because their daily use of medication. However, it has to be noted that total IgE is not a conclusive marker to determine the presence of allergic asthma, as therefore the measurement of specific IgE is more suitable.

From a mechanistic perspective, we have described earlier that activation of TLR2 is essential in the sitostanol induced Th1 shift [3]. The question is whether this finding could be linked to immunological aberrations in asthma. In this respect, it has already been shown that *in vitro* TLR2 stimulation reduced the production of Th2 cytokines, that play an important role in asthma, via the upregulation of Th1 cytokine production [34]. More recently, Aumeunier and co-workers [13] showed that TLR2 activation relieved asthmatic symptoms in mice. After an ovalbumine challenge in an experimental allergic situation in non-obese diabetic (NOD) mice, the addition of a TLR2 agonist reduced the asthmatic symptoms. In more detail, adding a TLR2 agonist to cells challenged with a house dust mite extract, which induces an allergic response (i.e. an increase in IL13 production), significantly reduced the HDM-induced production of IL-13. Moreover, Page et al [35] have shown that the Th2 mediated allergic response to a cockroach extract is significantly higher in TLR2(−/−) mice than in TLR2(+/+) wild type mice, also suggesting a protective effect of TLR2 on allergic responses. Altogether, stimulating TLR2 seems promising in relation to health improvement in asthma patients.

Interestingly, TLR2 activation is also known for its effects on Treg cell counts. Sutmuller and colleagues [36], [37] concluded that high concentrations of exogenous TLR2 ligand lead to increased proliferation of Treg cells, mostly under the influence of IL-2. In our experiments we found that sitostanol treatment showed a trend to higher Treg counts in PBMCs isolated from asthma patients. In line with the assumption of Sutmuller, we could also show an increase in IL-2, initiated by a TLR2 ligand – sitostanol – possibly explaining the increase in the number of Treg cells we observed.

The question arises what the role of regulatory T-cells in these changes in T helper cell behaviour is. It is known that this cell type is able to inhibit the activity of T-helper cells, when their response is dominant [38]. As such, the increased Treg cell counts and IL-10 production might fit the paradigm that sitostanol can rebalance the disturbed Th1/Th2 balance in asthma patients. Recently, also in humans the induction of Treg cells has been shown to be of importance in the treatment against asthma [20], [21], [39], [40], [41]. Moreover, the function of Treg cells is impaired in asthma patients [18], [42], and therefore an increase in IL-10 production seems by definition beneficial for this patient group. The involvement of IL-10 in the prevention of asthma and of IL-10 mutations in the onset of asthma is already well established in humans and mice [20], [21], [43], [44], [45]. In addition, Frossard and co-workers [46] showed in C3H/HeOuJ mice that IL-10 caused a decrease in allergic symptoms. Also, airway inflammation improved in BALB/c mice after IL-10 enhancement [47]. Valerio and co-workers [6] have shown an increased IL-10 production after a plant sterol enriched diet in a multiple sclerosis (MS) model in mice, leading to improved clinical manifestations of the disease. This is a strong indication that plant sterols and stanols indeed act via the Treg cells as MS is, in contrast to asthma, characterized by a more Th1 dominant immune response. Treg cells are also known to inhibit IL-17a production [15], which is a strong pro-inflammatory cytokine that plays an important role in airway inflammation in asthma patients [22], [48]. However, we did not find any effects of sitostanol on IL-17a production.

We exclude attribution of LPS contamination of used compounds to the observed effects, since blocking TLR4 has not influenced the results in previous experiments [3].

Interestingly, we found a significantly higher proportion of circulating NK cells in PBMCs from asthma patients at baseline. These cells play an important role in the regulation of airway eosinophilia in mice [49]. Despite the higher percentage we found in this study, it has been shown that NK cells from asthma patients have functional impairments, especially in evoking a Th1 like response upon dendritic cell stimulation [50], [51]. So it can be speculated that more cells are needed to guarantee a certain level of NK activity. Remarkably, we found a significant decrease in NK function after the addition of cholesterol to these cells. Little is known about the effects of cholesterol on asthma severity, but it has been shown that a high fat diet leads to increased airway inflammation post-prandially [52]. Moreover, the ability of cholesterol to inhibit NK function could explain the earlier described positive effects of statin therapy on asthmatic symptoms [53], [54]. The possible beneficial effects of statin treatment in asthma patients may also be due to a decrease in IL-17a production, caused by reduced serum cholesterol concentrations, as we here show that cholesterol incubation significantly increases IL-17a production, which is consistent with findings in mice that a high-fat diet leads to increased IL-17a production. However, findings on the effects of statins on asthma are far from consistent, as other groups have reported more severe asthma in statin users as compared to non-statin users [55], [56]. In contrast to cholesterol, sitostanol induced a significant decrease in the percentage of NK cells in the asthma group, but we did not find a change in NK cell function. This could be interpreted as if less NK cells are needed to maintain the necessary level of NK cell activity. However, this remains speculation. Furthermore, changes in the number of NK cells were, although statistically significant, very small and clinical relevance can be questioned, but needs in vivo verification.

In NKT cells we only found a trend towards a sitostanol-induced decrease in activity in cells from asthma patients as compared to cells from healthy controls. This again might be beneficial for these patients as the production of IL-4, IL-13 and IL-17a by these cells may play an important role in the onset and continuation of asthmatic responses [25], [26], [27]. However, it must be noted that degranulation activity is not per se similar to activity in cytokine production by these cells, as degranulation is a measure of kill-capacity of these cells and cytokine production might also be the result of an allergic reaction.

Interestingly, all effects described were found in PHA stimulated PBMCs, but not in HDM stimulated cells. We now speculate that the stimulation of these cells with a relatively high dose of purified HDM extract caused an acute allergic reaction, and it seems unlikely that a nutritional component could counteract this extremely strong immunological response. We presume that it is more likely, that a long-term exposure to sitostanol skews the T-helper cells more towards the Th1 side and therefore ultimately makes these cells less susceptible to respond to allergens in an *in vivo* situation, although *in vivo* proof for this assumption is certainly required.

**Figure 5:**
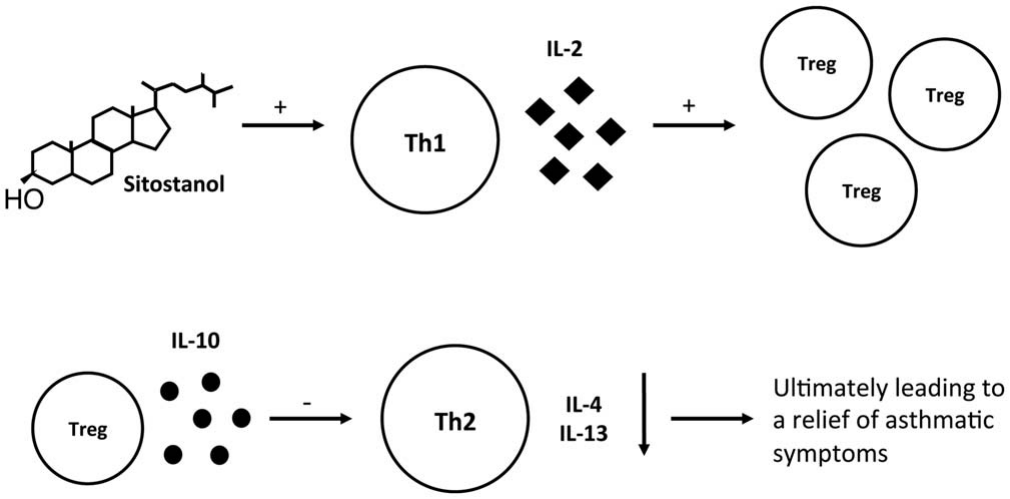
Based on the results of this pilot study we suggest the above pathway in which sitostanol induces IL-2 production in Th1 cells, which leads to the proliferation of Treg cells. These cells will produce higher amounts of IL-10, that can inhibit the activity of the dominant Th2 cells, which might ultimately lead to a relief of asthmatic symptoms.

In conclusion, we have shown that sitostanol is able to shift the disturbed Th1/Th2 response in PBMCs isolated from asthma patients into a more balanced response. Very likely, this works via an increase in Treg proliferation and activity. In combination with earlier findings concerning the role of TLR2 [3], [34], we now suggest a sitostanol-induced pathway as shown in figure 5, which ultimately results in a relief of allergic/asthmatic symptoms via increased Treg activity. Sitostanol activates TLR2 [3], inducing IL-2 production in Th1 cells. Il-2 is essential for Treg cell proliferation [36], leading to an increased IL-10 production. IL-10 has the ability to inhibit a Th2 dominant immune response and in mice and humans this has been shown to relieve asthmatic symptoms [20], [45], [46]. However, *in vivo* proof for this assumption demands specifically for this purpose designed double bind placebo controlled intervention studies in these patients.
